# Awareness and Work-Related Factors Associated with Scrub Typhus: A Case-Control Study from South Korea

**DOI:** 10.3390/ijerph15061143

**Published:** 2018-06-01

**Authors:** Dong-Seob Kim, Dilaram Acharya, Kwan Lee, Seok-Ju Yoo, Ji-Hyuk Park, Hyun-Sul Lim

**Affiliations:** 1 Department of Preventive Medicine, College of Medicine, Dongguk University, Gyeongju, 123 Dongdae-ro, Gyeongju-si 38066, Korea; cfs02@dongguk.ac.kr (D.-S.K.); dilaramacharya123@gmail.com (D.A.); kwaniya@dongguk.ac.kr (K.L.); medhippo@hanmail.net (S.-J.Y.); skeyd@naver.com (J.-H.P.); 2 Department of Community Medicine, Devdaha Medical College and Research Institute, Kathmandu University, Devdaha Municipality, Rupandehi 32900, Nepal

**Keywords:** awareness, scrub typhus, work related factors, South Korea

## Abstract

This study aimed to examine the awareness and the work-related factors associated with scrub typhus to provide data essential for evidence-based preventive strategies. A community-based case control study was carried out in the rural areas of Gyeongsangbuk-do, South Korea. Confirmed cases of scrub typhus (*n* = 57) were based on laboratory tests performed by the Korean Centers for Disease Control and Prevention (KCDC), 114 matched neighborhood controls, age (±6 years), gender and area of residence in the Gyeongsangbuk-do of South Korea. These cases were contracted over the 12-month period of January to December 2015. Overall, 61.4% cases and 79.8% of the control group had heard about scrub typhus. Cases were less aware about the fact that mites are mainly found in the bushes and that long sleeves and full-length pants and boots helped prevent scrub typhus. However, more were aware of the eschar lesion as a characteristic sign of scrub typhus. Work related risk factors such as having a wetland or puddles of water surrounding the house, dry field farming and working in the livestock industry were significantly associated with the scrub typhus. Health promotion strategies, such as creating general awareness, personal protection methods and improving personal hygiene and environmental sanitation in collaboration with relevant sectors, are recommended to reduce the burden of scrub typhus. Further intervention studies on awareness and behavioral and environmental modifications are required to investigate the effectiveness of such interventions.

## 1. Introduction

Scrub typhus is a mite-borne infection caused by *Orientia tsutsugamushi*. The disease is transmitted to humans through the bites of larvae of different species of trombiculid mites, appearing commonly in the autumn season [1,2]. Rodents provide a disease reservoir whereas mites act as a reservoir and a disease vector [3]. Clinical features at presentation range from non-specific flu-like symptoms such as fever, rash, an eschar at the bite site, headache, myalgia, and cough to severe systemic, life-threatening conditions involving the lungs, heart, liver, skin, central nervous system, and gastrointestinal tract [4,5]. Furthermore, it has been reported that inappropriately or untreated scrub typhus has a mortality rate of at least 30% [6].

Globally, one billion people are at risk of scrub typhus, which causes illness in one million people annually [1,7]. The endemicity of scrub typhus has been well reported in many Asian Pacific countries, including South Korea, Japan, China Taiwan, India, Indonesia, Thailand, Sri Lanka, Philippines, Australia, and the Western Pacific Islands [1,6,8,9]. In South Korea, the incidence of scrub typhus increased during from 2001 to 2013 where 70,914 cases were reported [10]. In 2016 alone, 11,105 patients were treated [11]. Observed increases in the incidence of scrub typhus in Laos, India, southern China, South Korea, and Japan may be due to improved diagnostic methods, medical investigation and awareness [12]. Moreover, a combination of climate change and the expansion of humans into previously uninhabited areas is likely to result in further increases in the incidence of the disease in near future [9,13,14].

Various previous studies have reported that scrub typhus in South Korea is more prevalent among those involved in agricultural work, females, and individuals aged ≥60 [15,16,17]. Given that scrub typhus is a mite borne disease, no man-to-man transmission occurs [18], but no licensed vaccine is available and no systematic vector control efforts are in place [19]. Thus, avoiding exposure to the vector [20] probably offers the best means of preventing and controlling the disease. Therefore, education about scrub typhus and work-related factors are an important aspect of health promotion strategies targeting disease control. This study aimed to document awareness and identify work-related factors associated with scrub typhus to provide data essential for evidence-based preventive strategies.

## 2. Materials and Methods

### 2.1. Ethics

This study was conducted after approval from Dongguk University, Gyeongju Hospital Clinical Examination Committee (110757-201406-HR-01-02, 110757-201503-HR-05-02). Written informed consent was obtained from all the subjects for participation in the study. All personal identifiers were removed before data analyses.

### 2.2. Study Design, Setting and Subjects

**Figure 1:**
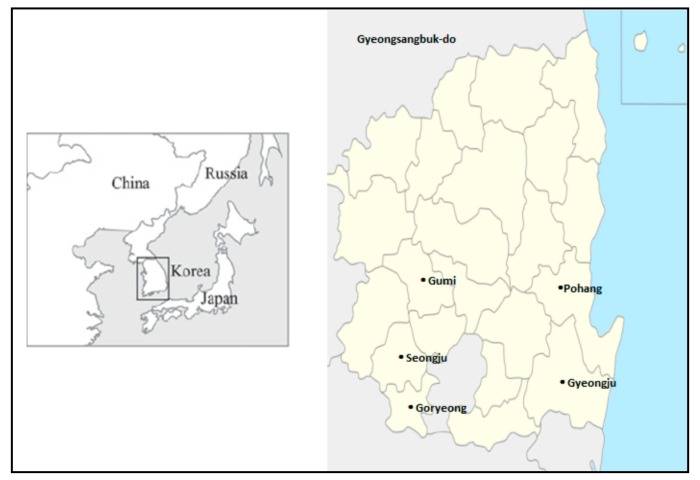
Location of selected study areas in Gyeongsangbuk-do Province of South Korea that were included in the case-control study.

This community-based case control study was conducted in five rural areas of Gyeongsangbuk-do (Gumi, Pohang, Seongju, Gyeongju, and Goryeong) of South Korea over a twelve-month period from January to December 2015. The target area was selected after requesting cooperation from all municipal and county public health centers in Gyeongsangbuk-do. Gyeongsangbuk-do is located between 35°34 E and 37°33 E north latitude. It lies in the southeastern region of the Korean peninsula and is bounded in the east by the East Sea, north by Gangwon-do and Chungcheongbuk-do, west by Chungcheongbuk-do and Jeollabuk-do, and south by Gyeongsangnam-do and Ulsan (Figure 1). As it is mostly surrounded by mountains, its climate is characterized by extremes both in summer and winter. The inland area is very hot and receives less rain than the other regions in summer. It is known as a center of agriculture, with 17.2% of the total population engaged in the agricultural business. Based on the natural environment and its biological resources, rice, apples, melons, and grapes are the major sources of agricultural production in the area. In addition to agriculture, the livestock industry (breeding cattle, goat, and chicken) is another major occupation of the people in this area [21,22]. We enrolled 57 scrub typhus cases and 114 controls (1:2) from the study area. The cases were identified using the legal reporting system for infectious diseases [15] within the past four weeks. In South Korea, physicians from public health center or private hospital reported every case of scrub typhus to the Korea Centers for Disease Control and Prevention (KCDC) through the National Notifiable Diseases Surveillance System (NNDSS). These reported cases are classified as “confirmed” based on one of the laboratory tests performed by KCDC:(i) an increase in the IFA IgM titer against O. tsutsugamushi to 1:16; (ii) an increase in the IFA IgG titer against *O. tsutsugamushi* to 1:256; and (iii) a fourfold increase in the indirect immunofluorescence assay (IFA) titer against *O. tsutsugamushi* [23].

Eligibility criteria for cases were: (i) confirmed cases of scrub typhus that were reported to Korean Centers for Disease Control and Prevention (KCDC) over a twelve-month period from January to December 2015; (ii) residents of Gyeongsangbuk-do (Gumi, Pohang, Seongju, Gyeongju, and Goryeong); and (iii) able to communicate and provide consent to participate in the study. The control group consisted of two neighbors, matched for age (±6 years), gender, and area of residence living nearest cases with no history of scrub typhus within 2 years as documented by the National Notifiable Disease Surveillance System (NNDSS) of South Korea [15]. In the case of unavailability of an eligible matched control in the nearest household, then the next nearest household was selected.

### 2.3. Data Collection

A letter containing the objectives of the study was sent to study participants requesting them to participate in the study. A standardized questionnaire was employed to collect information from study subjects. Trained interviewers visited cases and controls and conducted face-to-face interviews. The questionnaire used consisted of three parts: (i) baseline characteristics, (ii) awareness of scrub typhus, and (iii) work-related factors.

### 2.4. Definition of Variables

Baseline characteristics of the study participants were gender, age in years, education level, receipt of prevention education and awareness study by subjects-cases (before symptoms onset) and controls (before interview) and whether they had heard about scrub typhus. Gender was coded as male and female, age in years was categorized as: <50, 50–59, 60–69 and ≥70. Education level was coded as: none, primary school graduate (≤6 years of schooling), middle school graduate (6 to 9 years of schooling), high school graduate (9 to 12 years of schooling), college or university graduate (≥12 years of education). The degree of awareness was assessed using 16 closed questionnaires (scored on a yes/no basis) that were related to scrub typhus vector, the seasonality of scrub typhus, modes of transmission and preventive measures of scrub typhus. Similarly, 9 closed questions were asked to assess work-related risk factors of scrub typhus. These 9 questions were about: whether the participant’s house had a separate shower, wetland or puddle of water around the house, whether wild mice and rat secretion were seen during work; and the type of work engaged in. These included rice field related work such as cultivating a rice field, dry field farming, work related to cultivation of fruit trees, vinyl greenhouse farming and the livestock industry (breeding cattle, goat, and chicken).

### 2.5. Statistical Analysis

Data were entered using Microsoft Excel and analyses were conducted using Statistical Package for the Social Sciences (SPSS, IBM, Armonk, NY, USA), version 20.0. Firstly, the chi-square test was performed with independent factors of scrub typhus infection, and crude odds ratios (CORs) along with 95% confidence intervals (CI) were obtained. Second, the multivariate logistic regression model was employed to examine the awareness and work-related factors that were associated with scrub typhus infection. We entered in multivariate logistic regression model for all variables with significance level as: 0.15 ≤ *p* ≤ 0.20 [24] and a *p*-value < 0.05 was considered statistically significant.

## 3. Results

The baseline characteristics of cases and controls are detailed in Table 1. Of the 171 study subjects, 61.4% were females, 50.9% ≥70 years old, 25.4% had no education, and 90.1% had received no preventative education about scrub typhus. Overall, 61.4% cases (before symptoms onset) and 79.8% control (before interview) had heard about scrub typhus.

Case and control awareness of scrub typhus are summarized in Table 2. Controls provided more correct responses to items concerning scrub typhus such as the fact that mites are mainly found in bushes, the importance of wearing long sleeves, long pants, and boots to protect against scrub typhus. Additionally, participants were more aware than cases (*p* < 0.05) that they should avoid sitting or lying on grass, take off work clothes immediately after outdoor work, take a bath or shower immediately after working outdoors, keep work clothes and daily clothes separately. 

Work-related factors associated with scrub typhus are detailed in Table 3. Wetland or a puddle of water near the house (unadjusted odds ratio (UAOR) 2.60; 95% CI (1.19–5.65)) and work related to dry field farming (UAOR 2.46; 95% CI (1.19–5.06)) were found to be significantly associated with an increased risk of scrub typhus. 

The final logistic regression model is presented in Table 4. After adjusting for potential confounders, three components of disease awareness were found to be significantly associated with scrub typhus. Cases were less aware that mites are mainly found in bushes (AOR 0.14; 95% CI (0.03–0.58)) and of the need to wear work clothes that covered arms and legs with boots (AOR 0.08; 95% CI (0.013–0.47)). However, they were more aware that the most characteristic sign of scrub typhus is an eschar lesion (AOR 25.33; 95% CI (4.25–151.01)).

Adjusted multivariate analysis also showed the work-related factors such as a wetland or a puddle of water near home (AOR 2.87; 95% CI (1.09–7.51)), dry field farming (AOR 2.72; 95% CI (1.15–6.42)) and working with livestock industry (AOR 2.80; 95% CI (1.04–7.5)) were significantly associated with the increased risk of scrub typhus.

## 4. Discussion

Several good quality original and systemic review papers have been published recently on the epidemiological characteristics and clinical outcomes of scrub typhus, and without exception these studies show scrub typhus continues to be a serious public health problem in many endemic countries, including South Korea [3,6,10,25,26]. The present study addressed awareness and work-related factors associated with risk of scrub typhus using a community-based case control study design.

We found cases (before onset of symptoms) had heard about scrub typhus less commonly than controls (before our interview) and that controls were more aware of the disease than cases. This is consistent with the findings of a previous study conducted by Lee et al. in South Korea [27]. However, unlike the previous study, we found that females, individuals with a family history of scrub typhus and a history of receiving prevention education were significantly associated with scrub typhus awareness. We found some specific awareness components such as the knowledge that mites are mainly found in bushes, and that wearing work clothes that cover arms and legs with boots can protect from scrub typhus were significantly higher in controls than in cases. However, somewhat surprisingly, cases were more aware that the characteristic sign of scrub typhus is an eschar lesion probably because of their experience of the disease. These observations demonstrate health promotion strategies should focus on creating general awareness, improving personal hygiene, and emphasizing personal protection.

The other significant finding identified in the present study was that a wetland or a puddle of water near one’s house, dry field farming, and working with livestock are significant predictors of scrub typhus. These findings concur with those of a previous South Korean [28,29] and Chinese case control study [30] conducted in similar settings. The reason why a wetland and a puddle of water near home are risk factors of scrub typhus is that humidity and flourishing vegetation increase mite host densities [31]. In addition, since the subjects of the present study had jobs related to farming and livestock industry, transmission of scrub typhus might occur from domestic animal fodder, which attracts rodents [32,33,34]. Furthermore, those involved in dry field farming in South Korea are at high risk of contracting scrub typhus since *Leptotrombidium palladium*, which is a predominant mite species in South Korea, begins to appear in September and its population peaks in October–November [35]. Scrub typhus prevention strategies such as general cleanliness in living environments, reducing rat and mite numbers and personal protection should be recommended.

Our study has some definite strengths. First, we enrolled a wide geographic area representative of rural area population of Gyeongsangbuk-do, South Korea. Second, in the present study we utilized age, gender, and residential area matched neighborhood controls within the four weeks of duration, and therefore the chances of having recall bias might be minimal. Despite our efforts, the study has some specific limitations that should be sought in interpreting our study findings. First, we selected confirmed cases of scrub typhus as reported by the Korean Centers for Disease Control and Prevention (KCDC) that has been in use in South Korea as diagnostic criteria for scrub typhus cases [23]. Consequently, our methodology could not rule out the full details about the number of patients for where in vitro isolation, methodologies and antigenic strain for all diagnostic tests, and other methodical details in this regard. Moreover, KCDC did not have a use of polymerase chain reaction (PCR) of eschar swab to diagnose the scrub typhus, the most reliable indication of scrub typhus infection [36]. Although the choice of positivity cut-off titer for scrub typhus serologic testing varied by country and purpose of the IFA test, some scrub typhus hyperendemic countries have employed higher IgM IFA cut-offs [37,38]. Since our methodology made use of low IgM IFA diagnostic cut-offs, unintentional involvement of false positive cases might have distorted the study finding towards the null value and could be another weakness of the methodology section of this study. Further studies should understand the methodological disadvantages of the study. Secondly, its small sample size might affect the generalizability of our findings. Thirdly, our study population was from a community of farmers and livestock industry workers and therefore may not represent those living in urban areas. Lastly, the study did not address modes of transmission or cause and effect relationships. Nevertheless, findings could be useful for devising effective health promotion strategies targeting scrub typhus at the community level in endemic countries. Based on the findings of our study we can purpose some useful recommendations. Firstly, given that mite and rodent numbers are increased by climate warming [39], feasible strategies should be devised to minimize contact with vectors of scrub typhus. Furthermore, information in the form of education or educational materials concerning the prevention of scrub typhus has been shown to have a protective effect [27,28]. Therefore, we suggest health education guidelines be developed, community-based health awareness be increased, and environmental modifications be instituted in those regions where scrub typhus has been a long-standing public health problem. Secondly, we identified work-related predictors of scrub typhus contraction among individuals engaged in dry field and livestock industry, which we hope will aid the development of health promotion strategies in collaboration with governmental agricultural and veterinary departments.

## 5. Conclusions

Health promotion strategies need to place emphasis on creating general awareness, improving personal hygiene, methods of personal protection, and general cleanliness in living environments, and reducing number of rats and mites wherever possible in coordination with governmental agriculture and veterinary departments to reduce long-standing problems of scrub typhus in endemic countries. Further intervention studies on awareness and behavioral and environmental modifications are needed to investigate the effectiveness of such interventions.

## Figures and Tables

**Table 1:** Baseline characteristics of scrub typhus cases and controls.

Characteristics	Total *n* = 171 (%)	Case *n* = 57 (%)	Control *n* = 114	*p*-Value
Gender				1.000 *
Female	105 (61.4)	35 (61.4)	70 (61.4)	
Male	66 (38.6)	22 (38.6)	44 (38.6)	
Age (in years)				0.850 ^†^
<50	4 (2.3)	1 (1.8)	3 (2.6)	
50–59	34 (19.9)	11 (19.3)	23 (20.2)	
60–69	46 (26.9)	16 (28.1)	30 (26.3)	
≥70	87 (50.9)	29 (50.9)	58 (50.9)	
Education level				0.244 ^†^
None	43 (25.4)	11 (19.6)	32 (28.3)	
Primary school graduate(≤6 years of schooling)	61 (36.1)	29 (51.8)	32 (28.3)	
Middle school graduate (6 to 9 years of schooling)	30 (17.8)	9 (16.1)	21 (18.6)	
High school graduate (9 to12 years of schooling)	24 (14.2)	5 (8.9)	19 (16.8)	
College or university graduate (≥12 years of education)	11 (6.5)	2 (3.6)	9 (8.0)	
History of receiving prevention education about scrub typhus				0.366 *
No	154 (90.1)	53 (93.0)	101 (88.6)	
Yes	17 (9.9)	4 (7.0)	13 (11.4)	
Heard about scrub typhus				0.010 *
No	45 (26.3)	22 (38.6)	23 (20.2)	
Yes	126 (73.7)	35 (61.4)	91 (79.8)	

* by Chi-square test. ^†^ by Chi-square test for trend.

**Table 2:** Awareness of scrub typhus among cases and controls who were exposed within the previous one month in South Korea.

Awareness Components	Response	Total, 171 (%)	Case, 57 (%)	Control, 114 (%) ^†^	Crude OR * (95% CI)	*p*-Value
Occurs in Autumn (September–Novermber)	yes	87 (50.9)	23 (40.4)	64 (56.1)	0.52 (0.27–1.00)	0.052
Caused by bite with small mite	yes	76 (44.4)	22 (38.6)	54 (47.4)	0.69 (0.36–1.33)	0.27
Mites are mainly found in the bushes	yes	105 (61.4)	27 (47.4)	78 (68.4)	0.41 (0.21–0.79)	0.008
Symptoms of illness occur 1–2 weeks after bite by mite	yes	61 (35.7)	22 (38.6)	39 (34.2)	1.20 (0.62–2.33)	0.572
Symptoms are similar to cold symptoms (headache, fever and chills)	yes	103 (60.2)	34 (59.6)	69 (60.5)	0.96 (0.50–1.84)	0.912
The most characteristics sign is the eschar lesion	yes	83 (48.5)	32 (56.1)	51 (44.7)	1.58 (8.33–3.00)	0.160
Patient do not die when no treatment	yes	94 (55.0)	27 (47.4)	67 (58.8)	0.63 (0.33–1.19)	0.158
Does not infect others	yes	83 (48.5)	27 (47.4)	56 (49.1)	0.93 (0.49–1.76)	0.829
It does not re-occur once it is occurred	yes	51 (29.8)	15 (26.3)	36 (31.6)	0.77 (0.38–1.57)	0.478
We must wear long sleeves and pants work clothes and boots to prevent from scrub typhus	yes	120 (70.2)	33 (57.9)	87 (76.3)	0.42 (0.21–0.84)	0.013
Use of tick repellent prevents scrub typhus	yes	108 (63.2)	31 (54.4)	77 (67.5)	0.57 (0.29–1.10)	0.093
For prevention, should not sit or lie on the grass	yes	122 (71.3)	35 (61.4)	87 (76.3)	0.49 (0.24–0.98)	0.042
For prevention, do not put clothes on the grass	yes	121 (70.8)	35 (61.4)	86 (75.4)	0.51 (0.26–1.02)	0.057
For prevention, take off work clothes immediately after outdoor work	yes	121 (70.8)	34 (59.6)	87 (76.3)	0.45 (0.23–0.90)	0.024
For prevention, take a bath or shower immediately after working outdoors	yes	122 (71.3)	35 (61.4)	87 (76.3)	0.49 (0.24–0.98)	0.042
For prevention, keep work clothes and daily clothes separately	yes	123 (71.9)	35 (61.4)	88 (77.2)	0.47 (0.23–0.93)	0.030

* OR = odds ratio; CI = confidence interval; ^†^ Matched by age, gender, and residential area.

**Table 3:** Responses of cases and controls to work-related issues possibly associated with scrub typhus.

Work Related Factors	Response	Total, 171 (%)	Case, 57(%)	Control, 114 (%) ^†^	Crude OR * (95% CI)	*p*-Value
Residence house has separate shower	yes	152 (88.9)	54 (94.7)	98 (86.0)	2.93 (0.81–10.53)	0.085
A wetland or a puddle of water around the house	yes	33 (19.3)	17 (29.8)	16 (14.0)	2.60 (1.19–5.65)	0.014
Wild mice seen	yes	46 (26.9)	15 (26.3)	31 (27.2)	0.95 (0. 46-1.96)	0.903
Rat secretion seen	yes	40 (23.4)	16 (28.1)	24 (21.1)	1.46 (0.70–3.04)	0.307
Rice field related work (cultivating a rice field)	yes	71 (41.5)	25 (43.9)	46 (40.4)	1.15 (0.60–2.19)	0.661
Dry field farming	yes	110 (64.3)	44 (77.2)	66 (57.9)	2.46 (1.19–5.06)	0.013
Work related to cultivation of fruit trees	yes	22 (12.9)	10 (17.5)	12 (10.5)	1.80 (0.73–4.48)	0.196
vinyl greenhouse farming	yes	5 (2.9)	3 (5.3)	2 (1.8)	3.11 (0.50–19.17)	0.199
Livestock industry (breeding cattle, goat, and chicken)	yes	34 (19.9)	16 (28.1)	18 (15.8)	2.08 (0.96–4.47)	0.058

* OR = odds ratio; CI = confidence interval. ^†^ Matched by age, gender, and residential area.

**Table 4:** Associations between scrub typhus contraction and disease awareness and work-related factors as determined by multivariate analyses *.

Variables	Crude OR ** (95% CI)	*p*-Value	Adjusted_†_ OR ** (95% CI)	*p*-Value
Awareness				
Mites are mainly found in the bushes	0.41 (0.21–0.79)	0.008	0.14 (0.03–0.58)	0.006
The most characteristics sign is the eschar lesion	1.58 (8.33–3.00)	0.160	25.33 (4.25–151.01)	0.000
We must wear long sleeves and pants work clothes and boots to prevent from scrub typhus	0.42 (0.21–0.84)	0.013	0.08 (0.013–0.47)	0.005
Work related factors				
A wetland or a puddle of water around the house	2.60 (1.19–5.65)	0.014	2.87 (1.09–7.51)	0.032
Dry field farming	2.46 (1.19–5.06)	0.013	2.72 (1.15–6.42)	0.022
Livestock industry (breeding cattle, goat, and chicken)	2.08 (0.96–4.47)	0.058	2.80 (1.04–7.5)	0.041

* The significance level for entering the multivariate logistic regression model was set as 0.15 ≤ *p* ≤ 0.20 and a *p*-value < 0.05 was considered statistically significant; ** OR = odds ratio; CI = confidence interval.
